# The Anatomical Distribution of Trigeminal Neuralgia: A Perspective and Future Directions

**DOI:** 10.5812/kowsar.22287523.3641

**Published:** 2012-01-01

**Authors:** Peter M. Grace

**Affiliations:** 1 Department of Psychology and the Center for Neuroscience, University of Colorado at Boulder, Boulder, USA

**Keywords:** Trigeminal Neuralgia, Pain, Depression

Dear Editor,

Thank you for the invitation to present my views on the recent and very interesting clinical study by Bangash (1), wherein the location of trigeminal neuralgia (TN) among afflicted patients is described. It is very important that such clinical studies are conducted to characterize TN, for whilst the disease is relatively rare, it is nevertheless poorly understood, resulting in unsatisfactory treatment and profound suffering for the afflicted individual. Beyond these direct effects, TN, as with all types of chronic pain, is associated with other comorbidities, including depression, anxiety and drug abuse (2), as well as a significant economic burden to the health system and to society. The findings presented by Bangash (1) raise a number of interesting questions that warrant follow-up in additional studies, to aid in interpreting the results. The increased incidence of TN affecting the mandibular division in this patient population is an interesting finding, and Bangash (1) notes some potential reasons for this division being affected in particular. I believe it would also be worthwhile to raise an additional point regarding patient history. If the precipitating injury or stimulus were known, characterization would be worthwhile in order to determine whether there is anatomical overlap. Specifically, a literature is now forming around the idea that the immune system, microglia in particular, can be “primed” by a prior immune challenge, so that ensuing injury or disease (herpes zoster is a pertinent example for TN) can result exaggerated and long-lasting pain (3). Further studies involving a detailed analysis of patient history and the instance of damage to the affected nerve could potentially demonstrate clinical evidence to support these preclinical data. The prevalence of pain affecting the right side of the face is also very fascinating. Whether there is an underlying mechanism that requires exploration, such as immune priming, discussed above, or if it is peculiar to this patient cohort is not clear.

The other striking finding by Bangash (1) is that of the gender distribution of TN, which, as noted by the author, is in keeping with previous reports. Sex differences are fast becoming a topic of interest among basic and clinical scientists, and a recent study by Sorge and colleagues (4) has implicated immune mechanisms in this phenomenon. Additional phenotyping in this patient cohort may indeed show similar differences between males and females.

Finally, the study by Bangash (1) represents an important move towards mechanism-based diagnoses of chronic pain conditions (5). Anatomical location of the pain is a vital component to TN phenotyping, which will lead to a deeper understanding of the disease mechanisms operating in individual patients. Such an approach is likely to lead to improved treatment outcomes for all patients suffering from TN.
